# Enhancement of the RhoA/Rho kinase pathway is associated with stress‐related erectile dysfunction in a restraint water immersion stress model

**DOI:** 10.14814/phy2.15064

**Published:** 2021-10-21

**Authors:** Taiki Mori, Yuji Hotta, Daigaku Nakamura, Ryo Yahagi, Tomoya Kataoka, Kazunori Kimura

**Affiliations:** ^1^ Department of Hospital Pharmacy Graduate School of Pharmaceutical Sciences Nagoya City University Nagoya Japan; ^2^ Department of Clinical Pharmaceutics Graduate School of Medical Sciences Nagoya City University Nagoya Japan

**Keywords:** contraction, erectile dysfunction, rat, RhoA/Rho kinase pathway, stress

## Abstract

Stress is a risk factor for erectile dysfunction (ED); however, the pathology of stress‐induced ED remains unclear. Accordingly, in this study, we investigated the mechanisms of stress‐induced ED using a rat model. Ten‐week‐old male Wistar/ST rats were maintained in a cage filled with water to a height of 2 cm (stress group) or a normal cage (control group). We found that water immersion stress significantly enhanced the contractile response to noradrenaline in the corpus cavernosum (CC) (*p* < 0.05). Moreover, stress significantly decreased erectile function, as assessed by changes in intracavernous pressure (*p* < 0.01). In addition, Rho kinase‐1 (ROCK‐1) protein expression was significantly upregulated under stress conditions (*p* < 0.05), and phosphorylated myosin light chain (phospho‐MLC) levels, contribute to smooth muscle contraction, were also upregulated (*p* < 0.01). Treatment with fasudil hydrochloride, a Rho kinase inhibitor, for 5 days significantly improved erectile function (*p* < 0.01) and normalized ROCK‐1 and phospho‐MLC levels (*p* < 0.01). Thus, the RhoA/Rho kinase pathway may be associated with stress‐induced ED via contraction of CC. Stress also decreased the smooth muscle/collagen ratio of CC (*p* < 0.01), and fasudil treatment did not alleviate these effects (*p* = 0.50). These findings suggested that penile fibrosis gradually progressed under stress conditions and that fibrosis may be independent of the RhoA/Rho kinase pathway, implying that longer exposure to stress may promote ED. We conclude that stress‐induced ED was caused by contraction of CC mediated by the RhoA/Rho kinase pathway.

## Graphical abstract

Water immersion stress significantly enhanced contractile response in the corpus cavernosum (CC), decreased erectile function, and also upregulated RhoA/Rho kinase pathway in the CC. Fasudil, a Rho kinase inhibitor, improved erectile function. Our findings suggest that RhoA/Rho kinase pathway may be associated with stress‐induced erectile dysfunction via contraction of CC.

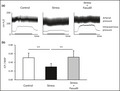

## 1 INTRODUCTION

Humans encounter much stress during daily life. In general, stressors can be divided into two categories: psychological stressors and physiological stressors. Psychological stressors are the negative feelings we experience under pressure, such as anxiety and depression. Physiological stressors are unpleasant factors that put strain on our bodies and can cause chronic pain and fatigue. In particular, fatigue is the most common physiological stressor among working men.

Physiological stress from fatigue can be a trigger in many diseases, including cardiovascular disease and gastritis. Erectile dysfunction (ED) is also caused by physiological stress. Huang et al. (2014) demonstrated that the prevalence rates of ED and asexuality status were 49.9% (*n* = 765) and 37.2% (*n* = 569) among 1531 men aged 40–80 years. In addition, the specific reasons for asexuality status in these individuals were severe stress (44.4%) and severe fatigue (26.3%). In other study, the incidence density rate of organic ED was 1.88‐fold higher in patients with chronic fatigue syndrome (CFS) than in patients without CFS (Chao et al., 2015). Thus, although physiological stress may be involved in ED, the mechanisms of stress‐induced ED remain unclear.

Penile erection is caused by corpus cavernosum smooth muscle (CCSM) relaxation, which results in increasing blood flow in CC. The nitric oxide/cyclic GMP pathway plays an important role in CCSM relaxation (Burnett et al., 1992; Ignarro et al., 1990). By contrast, stress activates the sympathetic nervous system, which results in smooth muscle contraction (Bianca et al., 2015; Martínez‐Martínez et al., 2014; Puzserova et al., 2012). There are two main pathways that contribute to smooth muscle contraction activated by the sympathetic nervous system. One mediates phospholipase C, which increases Ca^2+^ in smooth muscle cells, whereas the other mediates the RhoA/Rho kinase (ROCK) pathway, which is independent of Ca^2+^. Noradrenaline (NA), a neurotransmitter of sympathetic nerves, causes the exchange of GDP to GTP on the small GTPase RhoA via binding with the adrenaline α_1_ receptor (Liao et al., 2013). GTP‐RhoA activates ROCK, which inhibits myosin light chain (MLC) phosphatase and promotes the phosphorylation of MLC, resulting in smooth muscle contraction (Amano et al., 1996; Kimura et al., 1996). ROCK is present in two isoforms, that is, ROCK‐1 and ROCK‐2 (Ishizaki et al., 1996). The RhoA/ROCK pathway is associated with cardiovascular diseases via vascular smooth muscle contraction (Kureishi et al., 1997) and causes contraction of CC; enhancement of this pathway is associated with ED (Chitaley et al., 2001; Wang et al., 2002).

Therefore, we hypothesized that the RhoA/ROCK pathway may be involved in physiological stress‐induced ED. Accordingly, in this study, we investigated the mechanisms of stress‐induced ED, with a focus on the sympathetic nervous system, including the RhoA/ROCK pathway, using a rat model of physiological stress.

## 2 MATERIALS AND METHODS

### 2.1 Ethical approval

All experiments complied with the National Institutes of Health guidelines and were approved by the Institutional Animal Care and Use Committee of Nagoya City University (approval number: H25‐P‐09). Additionally, the experiments conformed to the principles and regulations described in *Journal of Physiology* (Grundy, 2015). All efforts were made to minimize animal suffering.

### 2.2 Animals

Experiments in this study were performed using 10‐week‐old male Wistar‐ST rats (Japan SLC Inc.). Animals were housed in an environmentally (temperature and humidity) controlled room with a 12‐h light/dark cycle and free access to laboratory chow and water.

### 2.3 Stress procedure

The stress model was based on a previously reported method (Tanaka et al., 2003). Rats were kept in cages filled with water to a height of 2 cm to induce physiological stress for five consecutive days. To keep the cages clean, all cages were replaced every day during the experiment.

### 2.4 Experimental design

**FIGURE 1:**
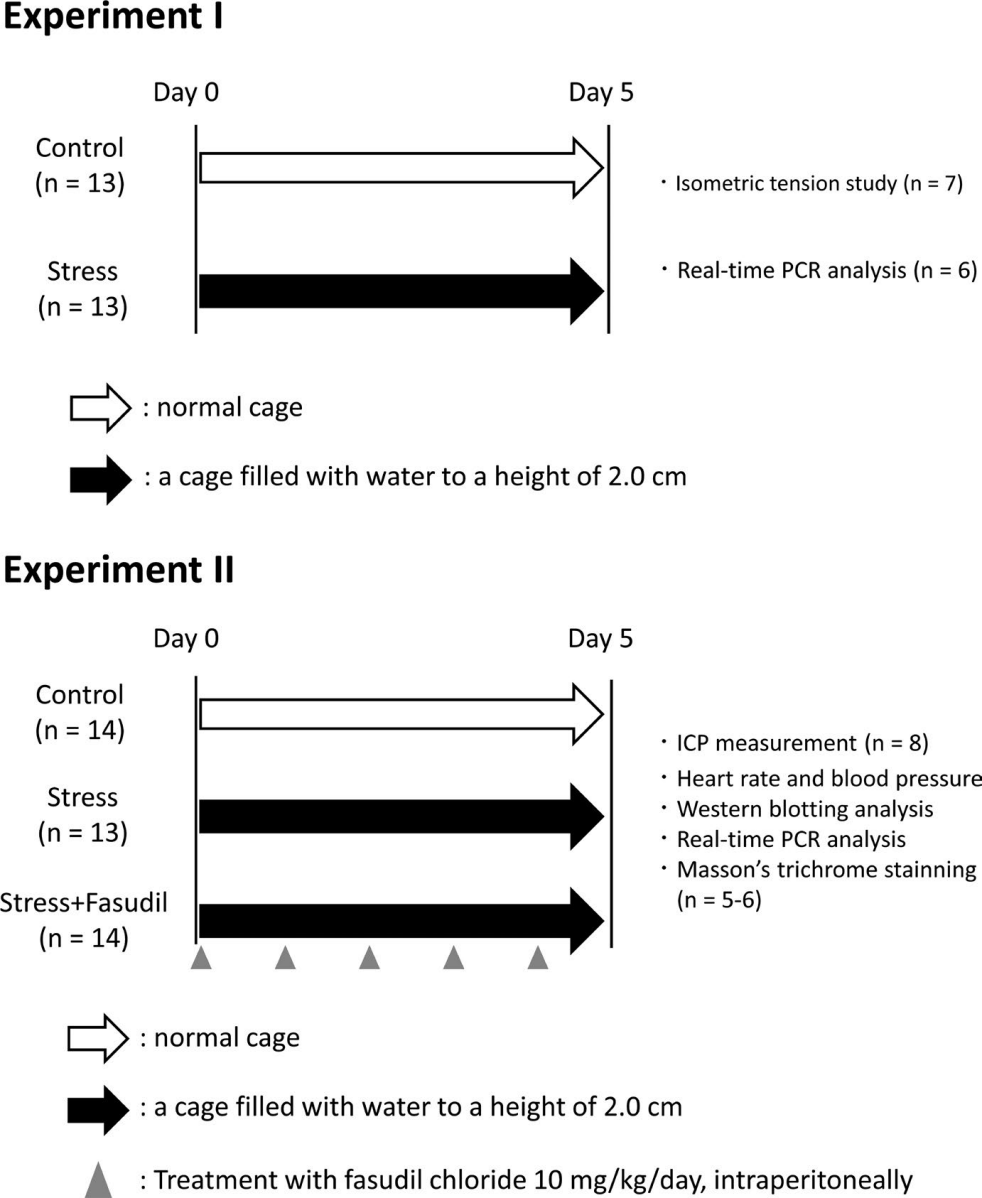
Experimental protocol

We performed two experiments, as illustrated in Figure 1. In experiment 1, the rats were divided into the control group (*n* = 13) and the stress group (*n* = 13). Rats in the control group were kept in normal cages for 5 days, whereas rats in the stress group were exposed to stress, as described above. Subsequently, penises were obtained from both groups of rats for analysis of isometric tension immediately after euthanasia (*n* = 7). Alternatively, penis samples were obtained from other rats for real‐time polymerase chain reaction (PCR) analysis (*n* = 6). The urethra, veins, and tunica albuginea were removed from penises, and CCs were used.

In experiment 2, the rats were divided into the control group (*n* = 14), stress group (*n* = 13), and stress+fasudil group (*n* = 14). The control and stress groups were maintained under the same conditions as described in experiment 1. Rats in the stress+fasudil group were treated with 10 mg/kg fasudil hydrochloride, a ROCK inhibitor (Tokyo Chemical Industry Co. Ltd) intraperitoneally once a day for 5 days during the stress period. Subsequently, the erectile function of rats in the three groups was evaluated as described below (*n* = 8).

Blood pressure was measured in another set of rats (*n* = 6). Penis samples were obtained soon after euthanasia, and CCs were used for western blotting and PCR analysis, whereas another portion of the penis was used for histological analysis. One rat in the stress group was excluded as an outlier.

### 2.5 Isometric tension study

Penis samples were obtained from rats under anesthesia. The urethra, veins, and tunica albuginea were removed from penises. The remaining CCs were prepared in a chilled Krebs solution composed of 119 mM NaCl, 4.6 mM KCl, 1.5 mM CaCl_2_, 1.2 mM MgCl_2_, 15 mM NaHCO_3_, 1.2 mM NaH_2_PO_4_, and 11 mM glucose. The CCs were suspended in an organ bath, which contained control solution (37℃), aerated with 95% O_2_ and 5% CO_2_. One side of the prepared CC was clipped, and the other side was ligated and connected to a force transducer (ADInstruments Pty. Ltd). The force transducers were connected to a bridge amp and PowerLab 4/26 (ADInstruments), and tension was measured using LabChart8 software (ADInstruments). The CCs were allowed to equilibrate for at least 1 h at an optimal resting tension of 0.6 g. After equilibration, the contractile responses to high KCl (80 mM) Krebs solution were measured to determine the reference contraction. Next, to investigate contractile responses, cumulative dose curves for NA (10^−10^–10^−4^ M; Sigma‐Aldrich Co. LLC) were measured, and the results were expressed relative to the responses to high KCl Krebs solution. Dose–response curve was fitted to all the data by nonlinear regression based on the Boltzmann model using the Origin software (OriginLab Corp.).

### 2.6 Real‐time PCR analysis

Total RNA was extracted from the CC using RNAiso plus reagent (Takara Bio Inc.), according to the manufacturer's recommended protocol. The RNA concentration and quality were measured via spectrophotometry at 260 and 280 nm using a Nanodrop 2000 (Thermo Fisher Scientific Inc.). Then, 1 μg of total RNA was reverse transcribed using ReverTra Ace (Toyobo Co. Ltd), dNTP mixture (Toyobo), RNase inhibitor (Toyobo), and oligo (dT)_20_ (Toyobo) in a TaKaRa PCR Thermal Cycler PERSONAL (Takara Bio). cDNA samples were diluted five times with RNase‐free water. Real‐time PCR was performed with KAPA SYBR green (KAPA Biosystems Inc.) according to the manufacturer's recommended protocol. All primer sequences are shown in Table 1. Initial denaturation was performed at 95℃ for 3 min using a CFX Connect Real‐Time PCR Detection System (Bio‐Rad Laboratories Inc.). Then, amplification was performed for 40 cycles of 95℃ for 3 s and 60℃ for 30 s. The specificity of each primer was confirmed by melt curve analysis. The data were analyzed using the ΔΔCt method, which indicates relative changes in gene expression normalized to the housekeeping gene, β‐actin.

**TABLE 1:** Primer sequences for real‐time quantitative polymerase chain reaction

Gene	Forward	Reverse
*α_1A_R*	5′‐CGAGTCTACGTAGTAGCC‐3′	5′‐GTCTTGGCAGTTTCTTC‐3′
*α_1B_R*	5′‐ATCGTGGCCAAGAGGACC‐3′	5′‐TTTGGTGCTTTCTTTTC‐3′
*ROCK‐1*	5′‐CTGGACATTTGAAGT‐3′	5′‐CCAACTGCTGTATCA‐3′
*ROCK‐2*	5′‐GAACCTACTCCTGGA‐3′	5′‐TGCTTCAGCAGCTCA‐3′
*TH*	5′‐TCGGAAGCTGATTGCAGAGA‐3′	5′‐TTCCGCTGTGTATTCCACATG‐3′
*TNF‐α*	5′‐GATCGGTCCCAACAAGGAGG‐3′	5′‐CTTGGTGGTTTGCTACGACG‐3′
β‐actin	5′‐TGTGTGGATTGGTGGCTCTATC‐3′	5′‐CATCGTACTCCTGCTTGCTGATC‐3′

Abbreviations: *ROCK*‐*1*, Rho kinase‐1; *ROCK*‐*2*, Rho kinase‐2; *TH*, tyrosine hydroxylase; *TNF*‐*α*, tumor necrosis factor‐α; α*_1A_R*, adrenaline α_1A_ receptor; *α_1B_R*, adrenaline α_1B_ receptor.

### 2.7 Evaluation of erectile function

Erectile function was assessed based on changes in intracavernous pressure (ICP), followed by electrostimulation of the cavernous nerves, as previously reported (Hotta et al., 2014). Briefly, under anesthesia with isoflurane (Pfizer Inc.), body temperature was maintained for each animal. First, polyethylene tubing (PE‐50) was inserted into the left carotid artery in order to record blood pressure. Next, the area posterolateral to the prostate was explored on both sides, and the major pelvic ganglions and cavernous nerves were identified and exposed. The skin overlying the penis was incised, the left penile crus was exposed, and a 23‐gauge needle connected to PE‐50 tubing filled with heparinized saline (5 U heparin/ml) was inserted into the crus for ICP measurement. Next, electrostimulation was performed using stainless steel bipolar wire electrodes (Unique Medical) and a pulse generator (Nihon Kohden) for cavernous nerve stimulation with the following parameters: 1 min at 5 V, 16 Hz, and a square wave duration of 5 ms. Systemic arterial pressure and ICP were recorded and analyzed with LabChart8 software (ADInstruments). Erectile function was evaluated using the maximum ICP to mean arterial pressure (MAP) ratio because ICP is influenced by systemic arterial pressure.

### 2.8 Western blot analysis

The penis samples were preserved at −80℃ immediately after collection from rats. The CC samples were homogenized in PRO‐PREP Protein Extraction Solution (iNtRON Biotechnology Inc.) with 1% Phosphatase Inhibitor Cocktail (Nacalai Tesque Co. Ltd) on ice, while paying attention to temperature change. Each protein sample was prepared strictly the same way. Protein (25 µg) was loaded into each lane and resolved by sodium dodecyl sulfate‐polyacrylamide gel electrophoresis. Proteins were subsequently transferred onto Immobilon‐P polyvinylidene difluoride membranes (Merck KGaA) using a Trans‐Blot SD Semi‐Dry Transfer Cell (Bio‐Rad). Membranes were blocked by treatment with 5% milk in Tris‐buffered saline containing 0.05% Tween 20 and probed with antibodies against the protein of interest as follows: anti‐ROCK‐1 (Abcam plc.; cat. no. ab45171; 1:5000 dilution), anti‐phospho (Ser‐19)‐MLC (CUSABIO Technology LLC; cat. no. P24844; 1:5000 dilution), and anti‐β‐actin (Sigma Aldrich; cat. no. A5316; 1:5000 dilution). Next, membranes were incubated with horseradish peroxidase‐conjugated anti‐mouse or anti‐rabbit secondary antibodies (GE Healthcare) and visualized using Immunostar ECL reagent (FUJIFILM Wako Pure Chemical Corp.) and an Amersham Imager 600 (GE Healthcare). Densitometry results were quantified using ImageJ (National Institutes of Health) and normalized to the expression of β‐actin. All results were expressed relative to the levels in control rats.

### 2.9 Histological analysis

Extirpated penises were fixed in 10% paraformaldehyde, which was replaced with 10%, 20%, and 30% sucrose solutions. The penis samples were then frozen in O.C.T. compound (Sakura Finetek Japan Co. Ltd). Seven‐micron cross‐sections were cut and used for Masson's trichrome staining. For quantitative image analysis, stained sections were photographed using an Eclipse Ti‐U instrument (Nikon Corp.) and NIS‐Elements D30 (Nikon). Saved images were analyzed using Photoshop CS4 (Adobe Systems Inc.). Red and blue pixel numbers in half of the CC tissue samples were analyzed for smooth muscle (SM) (red) and collagen (blue) staining, and the SM to collagen ratio was calculated.

### 2.10 Blood pressure measurement

We measured the blood pressure in each group of rats after the experimental period (day 5), using an indirect blood pressure meter (Softron Co. Ltd). The rats were kept in a tube with a heater in order to calm them down and keep them warm. After the rats were calmed, blood pressure was measured three times, and the results were averaged.

### 2.11 Statistical analysis

Results are expressed as means ± standard deviations. Outliers were excluded using Smirnov–Grubbs tests. For comparison of two groups, Student's *t*‐tests were used. For comparison of dose–response curve, repeated measures analysis of variance (ANOVA) was used. For comparisons of three groups, ANOVA was carried out. If the results of the ANOVA were significant, Bonferroni multiple *t*‐tests were used. Differences with *p* values <0.05 were considered significant.

## 3 RESULTS

### 3.1 Contractile responses of isolated CC samples

**FIGURE 2:**
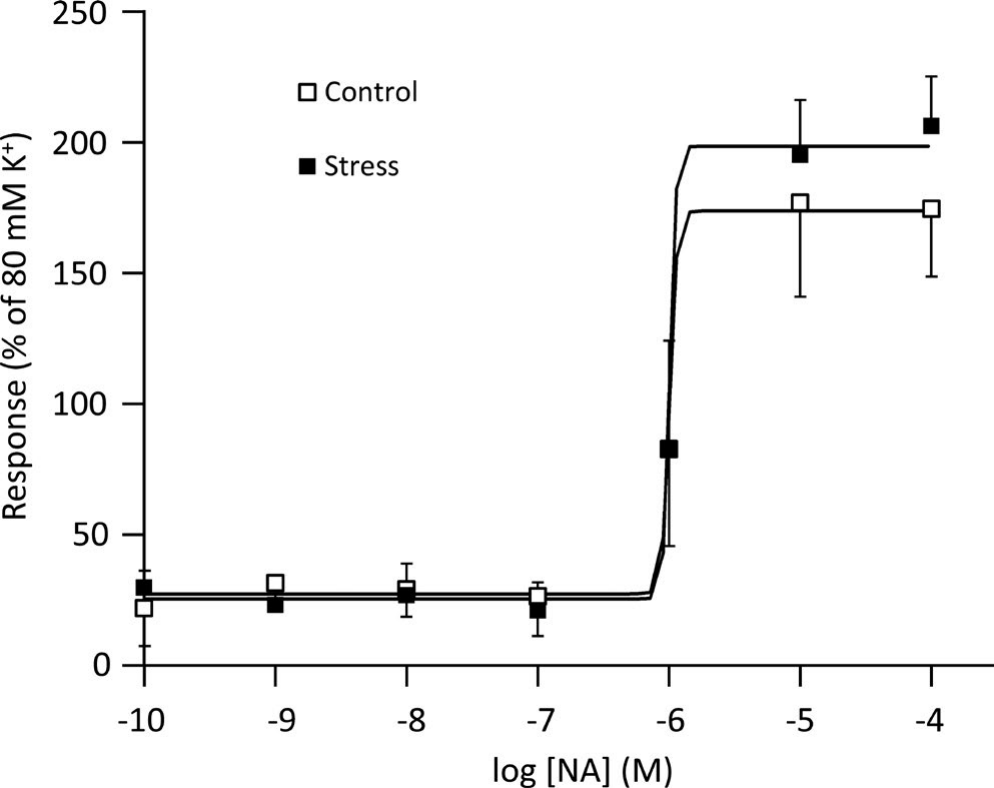
Cumulative dose responses to NA in isolated corpus cavernosum samples. The responses are relative to responses induced by 80 mM K^+^ Krebs solution. Curves were fitted to all the data by nonlinear regression. Data are reported as means ± standard deviations (*n* = 7/group). *p < 0.05 versus the control using Student's *t*‐tests. NA, noradrenaline

Figure 2 shows the cumulative dose–response curves to NA in isolated CC samples. The values of *E*max and EC_50_ were calculated (Table 2). The dose–response curve and the value of EC_50_ between two groups were not significantly different. However, the value of *E*max of the stress group was higher than that of the control group (*p* = 0.05).

**TABLE 2:** *E*max and EC_50_ for the responses to NA in isolated corpus cavernosum samples

	*E*max (% of 80 mM K^+^)	EC_50_ (M)
Control	176 ± 20	−5.86 ± 0.18
Stress	206 ± 27	−5.89 ± 0.22
*p*	0.05	0.8

Data are reported as means ± standard deviations (*n* = 7/group).

### 3.2 Expression levels of sympathetic nervous system‐related genes

**FIGURE 3:**
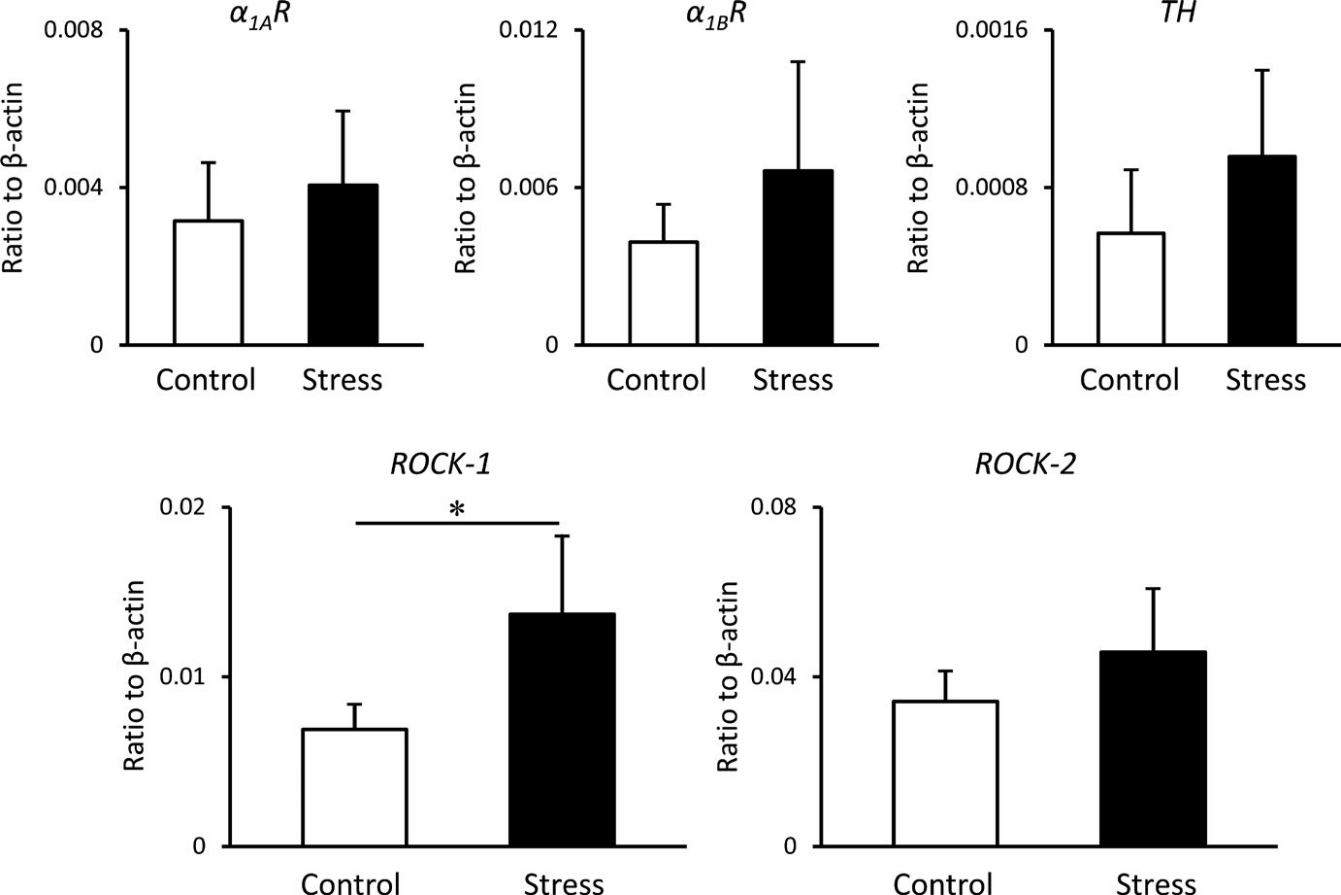
mRNA expression levels of sympathetic nervous system‐related genes in the rat corpus cavernosum. Target gene expression was quantified relative to the expression of β‐actin using the comparative cycle threshold method. Data are reported as means ± standard deviations (*n* = 6/group). *p < 0.05 using Student's *t*‐tests. TH: tyrosine hydroxylase, α_1A_R, adrenaline α_1A_ receptor; α_1B_R, adrenaline α_1B_ receptor; ROCK‐1, Rho kinase‐1; ROCK‐2, Rho kinase‐2

The mRNA expression levels of α_1A_ and α_1B_ adrenergic receptors in rat CCs in the stress group were higher than those in the control group (*p* = 0.41, *p* = 0.19, respectively); however, these results were not significant (Figure 3). Similarly, the mRNA expression levels of tyrosine hydroxylase, which is specifically expressed in sympathetic nerves, were increased in the stress group (*p* = 0.14), although the difference was not significant. Moreover, the mRNA expression levels of *ROCK*‐*1* in the stress group were significantly higher than those in the control group (*p* < 0.05). Although *ROCK*‐*2* mRNA levels were also increased in the stress group, the result was not significant (*p* = 0.14).

These results suggest that stress caused the contraction of the CC via the RhoA/ROCK pathway. Therefore, we exposed another group of rats to water immersion‐restraint stress and treated the rats with fasudil hydrochloride (stress+fasudil group) to further investigate whether stress caused ED via the RhoA/ROCK pathway.

### 3.3 Changes in ICP during electrostimulation

**FIGURE 4:**
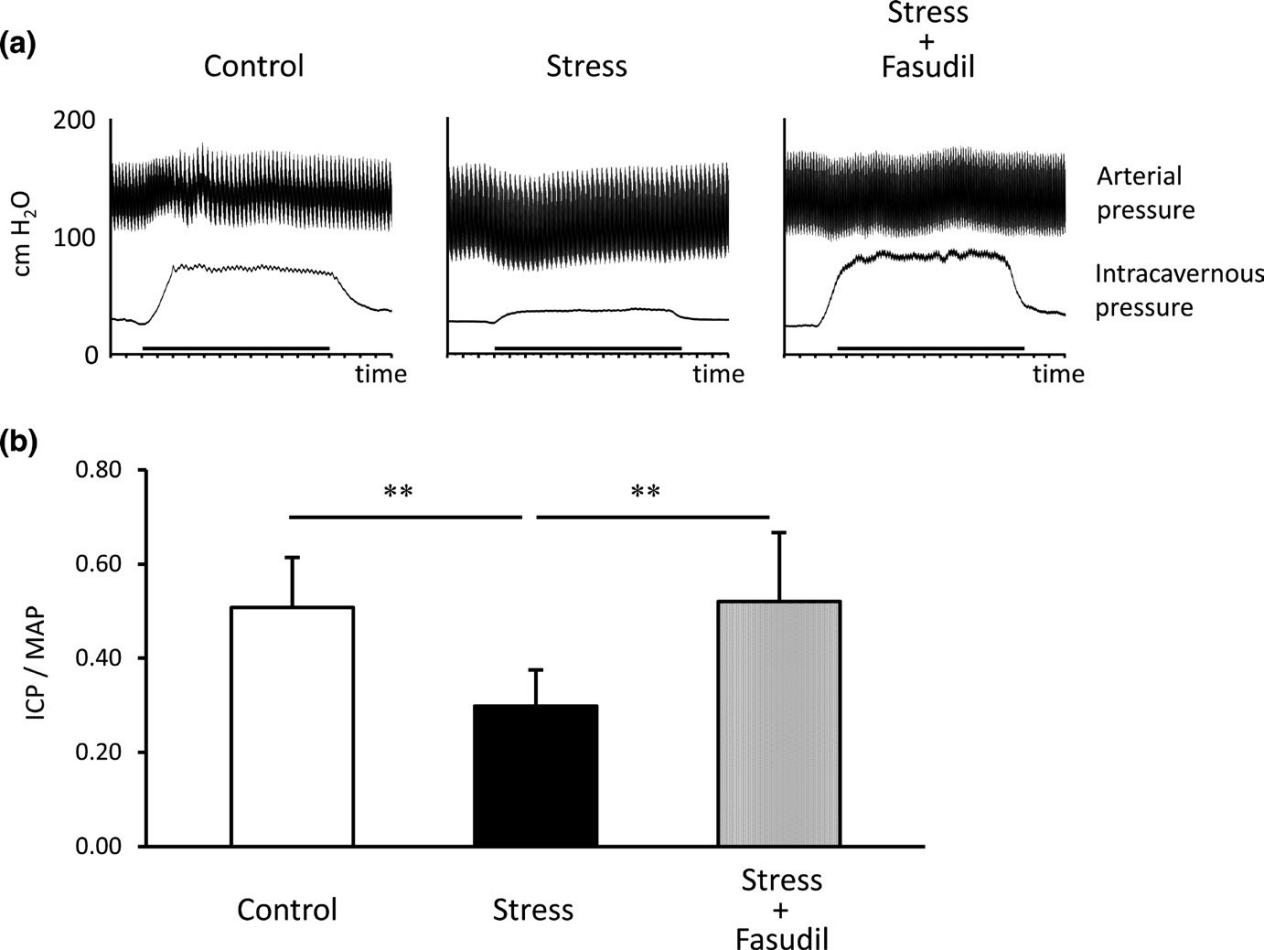
Representative ICP and AP results. Charts show ICP and AP during electrostimulation (bold bar) in each group of rats (a). The EFS period is shown by the thicker bar. ICP to MAP ratios in each group rats were measured (b). Data are reported as means ± standard deviations (*n* = 8/group). **p < 0.01 using Bonferroni's multiple *t*‐tests. ICP, intracavernous pressure; MAP, mean arterial pressure

Figure 4a shows the representative ICP charts for each group rats. The ICP to MAP ratios in the stress group were significantly lower than those in the control group (stress group, 0.30 ± 0.03 vs. control group, 0.51 ± 0.04, *p* < 0.01; Figure 4b). However, those in the stress+fasudil group were significantly higher than those in the stress group (stress+fasudil group, 0.52 ± 0.05 vs. stress group, 0.30 ± 0.03, *p* < 0.01).

### 3.4 Expression levels of RhoA/ROCK pathway‐related proteins

**FIGURE 5:**
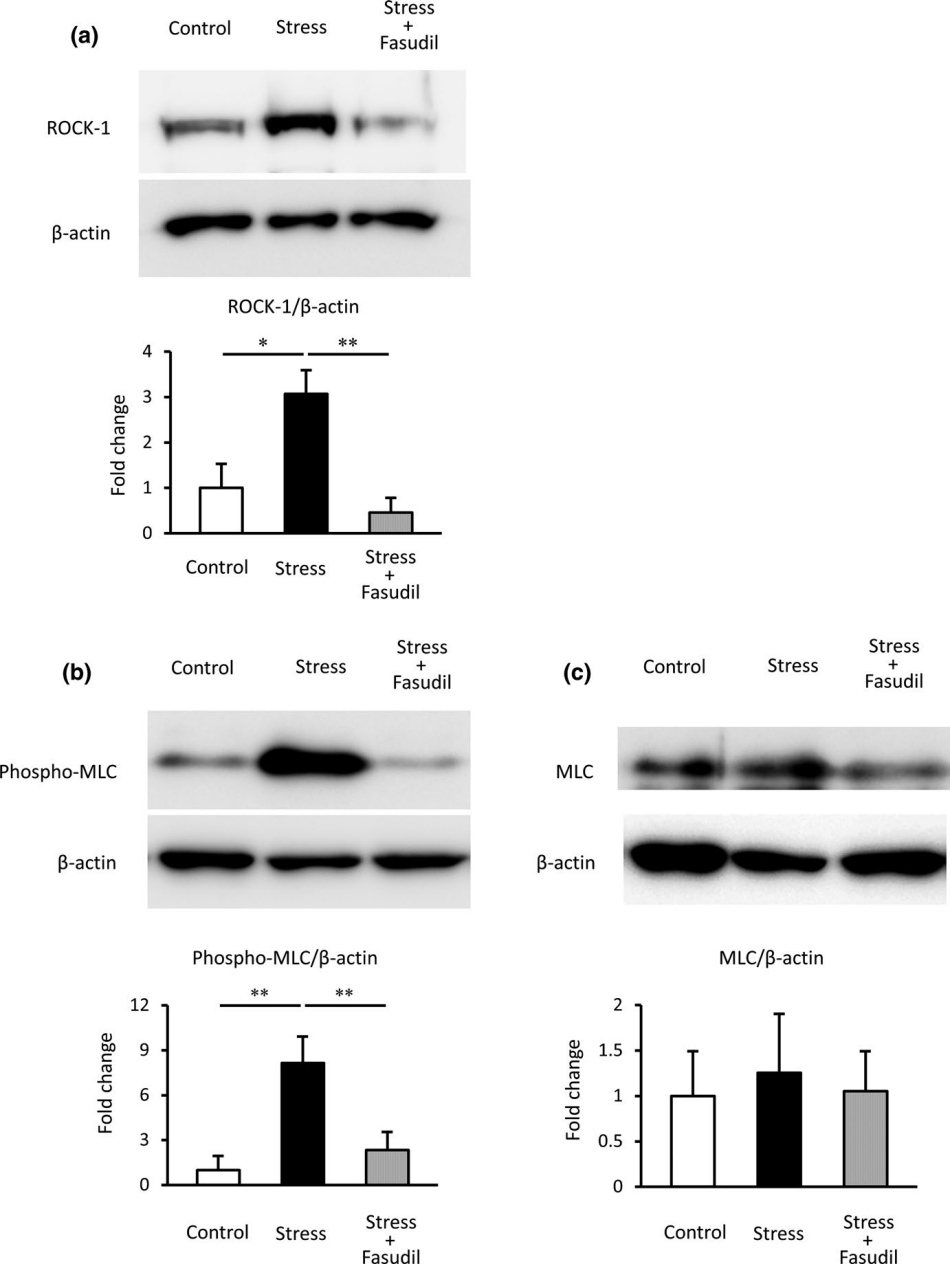
Protein levels of ROCK‐1 (a) and phospho‐MLC (b) in rat corpus cavernosum. Quantification was performed by densitometry analysis, with normalization to β‐actin. Data are expressed as the ratio compared with the control. Data are reported as means ± standard deviations (*n* = 5–6/group). *p < 0.05, **p < 0.01 using Bonferroni's multiple *t*‐tests. ROCK‐1, Rho kinase‐1; phospho‐MLC, phosphorylated‐myosin light chain

The protein expression levels of ROCK‐1 in the stress group were significantly higher than those in the control group (*p* < 0.05; Figure 5a), whereas those in the stress+fasudil group were significantly lower than those in the stress group (*p* < 0.01). Next, we evaluated activated MLC by measuring the levels of phosphorylated myosin light chain (phospho‐MLC) protein. The levels of phospho‐MLC in the stress group were also significantly higher than those in the control group (*p* < 0.01; Figure 5b). Moreover, the levels of phospho‐MLC in the stress+fasudil group were significantly lower than those in the stress group (*p* < 0.01). We also evaluated total MLC expression. The protein expression levels of total MLC were not significantly different among the three groups (Figure 5c).

### 3.5 SM/collagen ratio of CC

**FIGURE 6:**
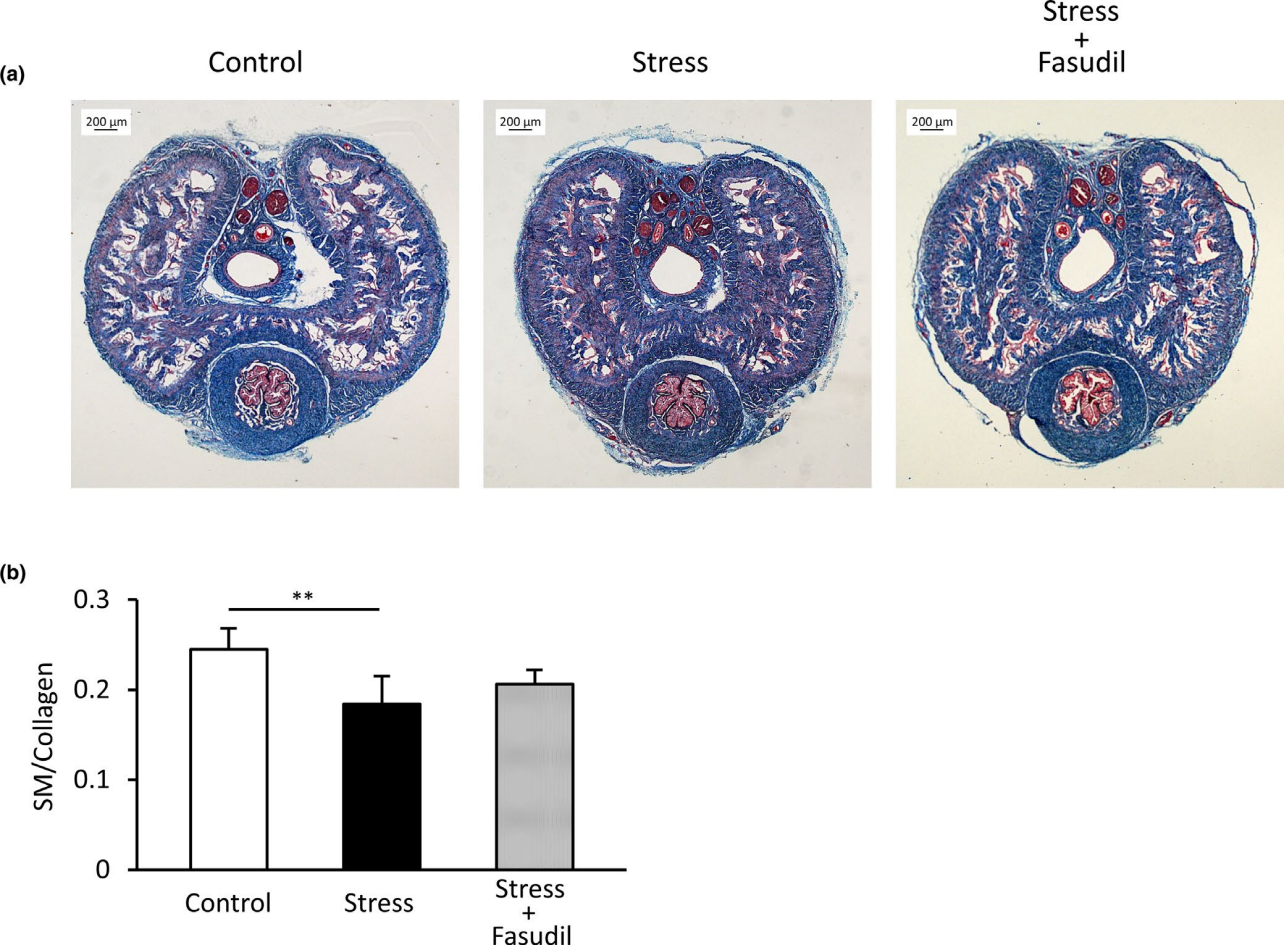
Histological analysis of the rat corpus cavernosum. Representative rat penises stained with Masson's trichrome stain (a). SM is stained red and collagen is stained blue. SM/collagen ratios in the rat corpus cavernosum were evaluated (b). Data are reported as means ± standard deviations (*n* = 5–6/group). **p < 0.01 using Bonferroni's multiple *t*‐tests. SM, smooth muscle

Figure 6a shows histological images in each group of rats and Figure 6b shows the SM/collagen ratio in each group of rats. The SM/collagen ratio in the stress group was significantly lower than that in the control group (*p* < 0.01). Treatment with fasudil did not significantly improve the SM/collagen ratio (*p* = 0.50).

### 3.6 mRNA expression levels of inflammatory cytokines

**FIGURE 7:**
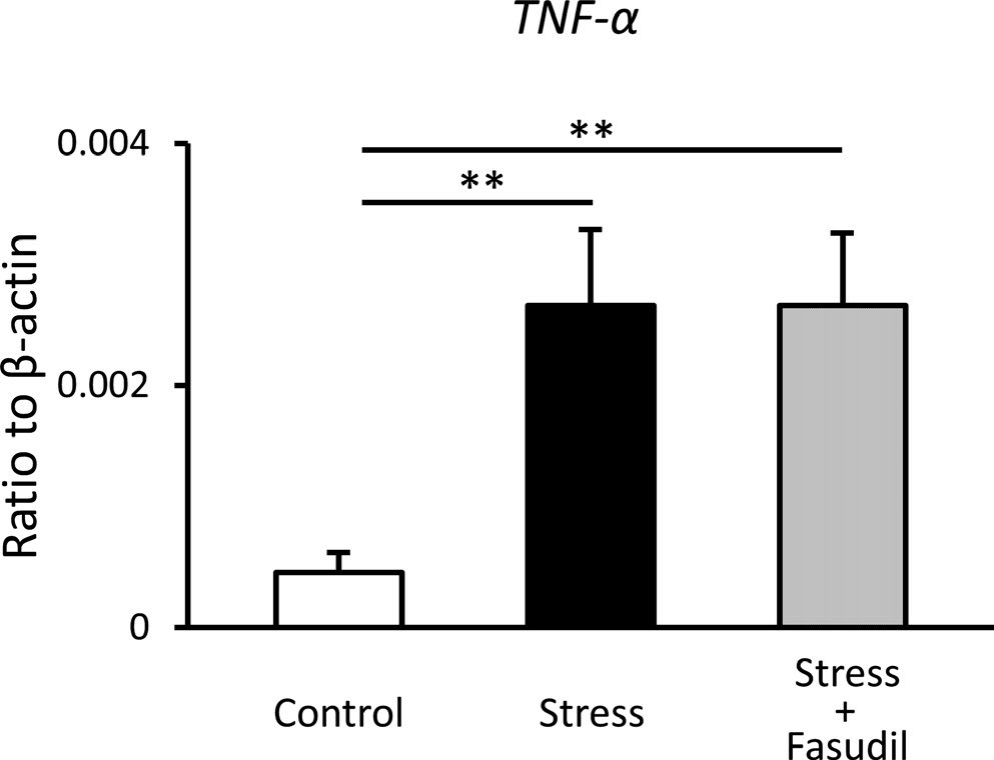
mRNA expression levels of inflammatory cytokines in the rat corpus cavernosum. Target gene expression was quantified relative to the expression of β‐actin using the comparative cycle threshold method. Data are reported as means ± standard deviations (*n* = 5–6/group). *p < 0.05, **p < 0.01 using Bonferroni's multiple *t*‐tests. *TNF*‐*α*, tumor necrosis factor‐α

The mRNA expression levels of tumor necrosis factor (*TNF*)‐*α* in the stress group were significantly higher than those in the control group (*p* < 0.01; Figure 7). Additionally, *TNF*‐*α* levels in the stress+fasudil group were significantly higher than those in the control group (*p* < 0.01). There were no differences between the stress group and the stress+fasudil group.

### 3.7 Heart rate and blood pressure

As shown in Table 3, the heart rate in the stress group was significantly higher than that in the control group (*p* < 0.01). Moreover, the heart rate in the stress+fasudil group was not changed compared with that in the stress group, and significantly higher values were maintained compared with those in the control group. However, blood pressure values did not differ significantly among the three groups.

**TABLE 3:** Heart rate and systemic blood pressure

	HR (bpm)	SBP (mmHg)	MBP (mmHg)	DBP (mmHg)
Control	356.4 ± 24.2	104.8 ± 2.4	88.3 ± 2.3	80.1 ± 2.1
Stress	476.8 ± 11.4**	98.7 ± 3.4	83.1 ± 3.2	75.4 ± 3.2
Stress+fasudil	463.7 ± 6.8**	96.1 ± 3.3	80.3 ± 2.7	72.6 ± 2.5

Data are reported as means ± standard deviations (*n* = 5–6/group).

Abbreviations: HR, heart rate; SBP, systolic blood pressure; MBP, mean blood pressure; DBP, diastolic blood pressure.

** *p* < 0.01 versus the control group using Bonferroni's multiple *t*‐tests.

## 4 DISCUSSION

In this study, we aimed to clarify the mechanisms of stress‐induced ED using a rat model of water immersion‐restraint stress. We found that water immersion stress augmented the maximum contractile response to NA in rat CC samples. Moreover, stress decreased erectile function and enhanced protein levels of ROCK‐1 and phospho‐MLC, but did not change protein levels of total MLC. We also showed that fasudil, a ROCK inhibitor, improved erectile function and normalized the levels of ROCK‐1 and phospho‐MLC. Therefore, our findings suggested that the RhoA/ROCK pathway was associated with stress‐induced ED via augmentation of CC contraction. Some studies have reported that the RhoA/ROCK pathway is associated with ED. For example, Hannan et al. (2013) revealed that the RhoA/ROCK pathway has detrimental effects on erectile function after bilateral cavernous nerve injury (BCNI) and that ROCK inhibition alleviates ED associated with BCNI by preserving penile nitric oxide bioavailability and decreasing penile apoptosis. Additionally, Sezen et al. (2014) showed that erectile response was significantly reduced in diabetic rats compared with that in nondiabetic rats and was preserved in diabetic rats treated with hydroxyl fasudil. In this study, we demonstrated that the RhoA/ROCK pathway may be also involved in stress‐induced ED in a rat model of fatigue‐stress for the first time. In addition, treatments targeting the RhoA/ROCK pathway may be effective for stress‐induced ED.

Our current histological analysis showed that penile fibrosis gradually progressed under stress conditions. Interestingly, although fasudil treatment improved erectile function, penile fibrosis caused by stress was not inhibited. Thus, our findings suggested that penile fibrosis may be independent of the RhoA/ROCK pathway under stress conditions and may be caused by inflammation. Indeed, we found that the mRNA levels of *TNF*‐*α*, an inflammatory cytokine, were upregulated by stress in the CC. A previous study showed that serum inflammatory cytokine levels were increased in both acute or chronic psychological stress conditions (Himmerich et al., 2013). Moreover, there is a correlation between the inflammatory response and penile fibrosis. Serum inflammatory cytokine levels are known to be elevated, and the SM/collagen ratio is decreased in the CC of a rat model of prostatitis (Hu et al., 2016). Therefore, stress may promote penile fibrosis via production of inflammatory cytokines.

In this study, systemic blood pressure did not differ among the three groups. Thus, stress and fasudil treatment both had no effect on systemic blood pressure. However, there were some limitations to this study. As previously reported, relaxation of the CC plays important roles in achieving a complete erection (Andersson, 2001). Here, we focused on the contractile system and did not investigate the effects of fatigue‐stress on the relaxing system in the CC. Thus, further studies are needed to better identify the mechanisms.

In this study, 10‐week‐old rats were used. It has been reported that it is not acceptable to relate human and rat age by simply correlating their life span because human age in years and rat age in days differ depending on the phase of life. Furthermore, it has been reported that sexual maturity occurs at about the 6th week in male rats and that 10‐week‐old rats are approximately equivalent to young adult humans (Sengupta, 2013).

In conclusion, we found that physiological stress caused ED via the RhoA/ROCK pathway for the first time. ROCK inhibitors may be useful for stress‐induced ED. Accordingly, relieving stress early may be the most important factor for avoiding organic ED.

## 5 CONCLUSION

In this study, we found that physiological stress caused ED via enhancement of CC contraction, which was mediated by the RhoA/ROCK pathway. Treatment targeting the RhoA/ROCK pathway may be effective for stress‐induced ED. However, penile fibrosis could be caused by stress conditions independent of the RhoA/ROCK pathway.

## CONFLICT OF INTEREST

The authors have no conflict of interest relevant to this article.

## AUTHOR CONTRIBUTIONS

T.M., Y.H., and K.K. conceived and designed the study; T.M., Y.H., D.N., and R.Y. contributed for the acquisition of data. T.M., Y.H., and T.K. analyzed the data. T.M., Y.H., and K.K. drafted the article and approved the final version of the paper. All authors approved the manuscript and agreed to be accountable of the manuscript.
